# Pigmentation and Spectral Absorbance Signatures in Deep-Water Corals from the Trondheimsfjord, Norway

**DOI:** 10.3390/md10061400

**Published:** 2012-06-20

**Authors:** Anette C. Elde, Ragnhild Pettersen, Per Bruheim, Johanna Järnegren, Geir Johnsen

**Affiliations:** 1 Trondheim Biological Station, Department of Biology, Norwegian University of Science and Technology (NTNU), Trondheim NO-7491, Norway; Email: anette.c.elde@gmail.com (A.C.E.); ragnhild.pettersen@bio.ntnu.no (R.P.); 2 Department of Bio-Technology, Norwegian University of Science and Technology (NTNU), Sem Sælands vei 8, Trondheim NO-7491, Norway; Email: per.bruheim@biotech.ntnu.no; 3 Norwegian Institute for Nature Research (NINA), Trondheim NO-7485, Norway; Email: Johanna.Jarnegren@nina.no; 4 The University Centre on Svalbard (UNIS), P.O. Box 156, Longyearbyen NO-9171, Norway

**Keywords:** astaxanthin, canthaxanthin, optical characteristics, *Lophelia pertusa*, *Paragorgia arborea*, *Primnoa resedaeformis*

## Abstract

The pigmentation and corresponding *in vivo* and *in vitro* absorption characteristics in three different deep-water coral species: white and orange *Lophelia pertusa*, *Paragorgia arborea* and *Primnoa resedaeformis*, collected from the Trondheimsfjord are described. Pigments were isolated and characterized by High-Performance Liquid Chromatography (HPLC) analysis and High-Performance Liquid Chromatography Time-Of-Flight Mass Spectrometer (LC-TOF MS). The main carotenoids identified for all three coral species were astaxanthin and a canthaxanthin-like carotenoid. Soft tissue and skeleton of orange *L. pertusa* contained 2 times more astaxanthin g^−1^ wet weight compared to white *L. pertusa*. White and orange *L. pertusa* were characterized with *in vivo* absorbance peaks at 409 and 473 nm, respectively. *In vivo* absorbance maxima for *P. arborea* and *P. resedaeformis* was typically at 475 nm. The shapes of the absorbance spectra (400–700 nm) were species-specific, indicated by *in vivo*, *in vitro* and the corresponding difference spectra. The results may provide important chemotaxonomic information for pigment when bonded to their proteins *in vivo*, bio-prospecting, and for *in situ* identification, mapping and monitoring of corals.

## 1. Introduction

Deep water corals have a cosmopolitan distribution and are found in almost all of the world’s oceans. The scleractinian (stony coral) *Lophelia pertusa* is a reef forming coral found at depths between 39 and 3383 m, but most commonly occurs between 200 and 800 m [1,2]. This species creates large reef complexes of great ecological importance by providing shelter, food and substrate for a variety of other species. In the north-east Atlantic, which houses the main part of the *L. pertusa* reefs discovered in the world to date [2], more than 1300 species have been registered that are associated with the reefs, which is equivalent to the diversity of tropical shallow water reefs [1]. Deep-water coral reefs are dispersed along the Norwegian coast, and the Røst reef (approximately 67°19′ N; 9°02′ E) southwest of Lofoten is the largest known *L. pertusa* reef in the world [3]. *Paragorgia arborea* and *Primnoa resedaeformis* are deep water gorgonian corals with a soft skeleton that with their large and branching morphology also create habitats of importance to other species [4,5,6]. They occur on both sides of the North Atlantic, most commonly at depths between 200 and 1000 m [7,8,9].

Deep-water corals are passive suspension feeders [2] using their polyps to capture zooplankton [10,11,12,13] and they also feed on dead particulate matter [14]. They are azooxanthellate organisms [1,15] living primarily below the euphotic zone, yet many of these organisms are as intensely colored as tropical corals. Color is defined as the reflection of different wavelengths of visible light, hence coloration is the result of selective absorption [16]. *Lophelia pertusa* has two basic color morphologies; orange and white [17]. *Paragorgia arborea* individuals vary from deep red to white-pink, whereas white individuals also exist. *Primnoa resedaeformis* do not show as much color variation, and is usually orange-yellow [5]. The color variation between these species is likely due to carotenoids, probably bound to specific proteins. 

Carotenoids generate bright coloration in several taxa, such as birds, insects, fish and crustaceans [18], and are also present in many marine invertebrates and terrestrial animals [19]. The carotenoids are produced in photosynthetic bacteria, higher plants, micro- and macroalgae [20] and are further metabolically transformed through the food web into deep-water corals examined in this study. Altogether, more than 600 different carotenoids exist [21] and in marine phytoplankton more than 30 major carotenoids can be found [22,23]. Carotenoids serve different purposes in marine organisms, e.g., light absorption and utilization in photosynthesis and photoprotection in algae, and as camouflage [19] in deep sea living organisms. Carotenoids also function as antioxidants [24] which possibly may contribute to the strengthening of coral immune systems. In deep-water corals, the carotenoids (metabolites) may provide an antibacterial function, as the corals secrete mucus that contains antibacterial substances to remove sediments and particles [25].

One of the major carotenoids found in marine invertebrates is astaxanthin [16,26]. Since animals are unable to synthesize carotenoids *de novo* [27] they obtain these pigments from their diet, *i.e.*, the absorbed carotenoids are transformed into other carotenoid derivatives [23,28]. It has been suggested that organisms produce different forms of carotenoids, and the majority of these are oxidation products of β,β-carotene. The metabolic pathway is different for most organisms, however the end product, astaxanthin, is often the same [29]. Carotenoids do not usually appear in free form in organisms, and can emerge as carotenoproteins or be bound to esters, glycosides and sulfates [19]. Carotenoids can also, as in scleractinian corals, be bound to calcium carbonate. The physical and chemical properties of the carotenoid can be changed when bound to proteins or other molecules, and can cause a spectral shift in the light absorption spectrum [24]. *In vivo* and *in vitro* absorption spectra (400–800 nm) give an indication of which major pigments are present in corals. Only one publication [30] related to pigment composition in *L. pertusa*, *P. arborea* and *P. resedaeformis* is known to the authors.

The main objective of this study was to determine pigmentation and corresponding optical signature using *in vivo* and *in vitro* absorption characteristics in *P. arborea*, *P. resedaeformis* and two color morphs of *L. pertusa*. *In vivo* bio-optical characteristics from coral species pigments may provide important chemotaxonomic information (pigments bonded to their specific proteins making specific signatures), future identification of pigments for bio-prospecting and for *in situ* identification, mapping and monitoring of corals.

## 2. Results and Discussion

**Figure 1:**
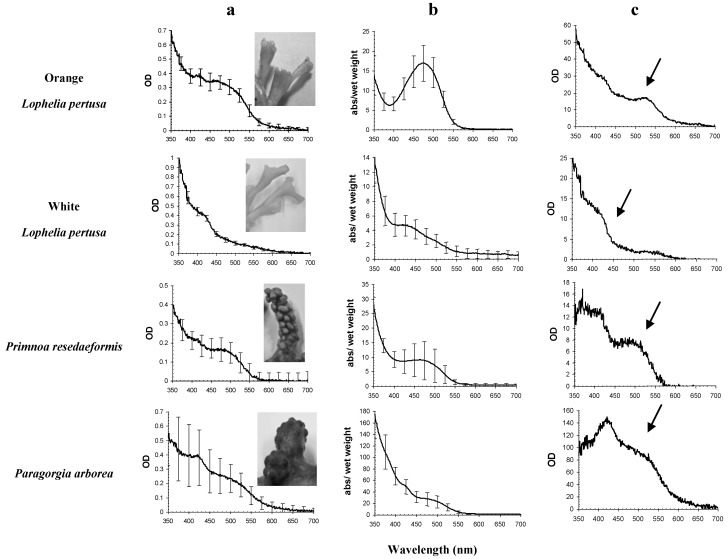
Pigment absorption signatures in all coral species, *in vivo* absorbance (column **a**) and *in vitro* weight specific (g^−1^ wet weight) absorption curves in acetone raw extracts (column **b**), and the corresponding difference spectra between **a** and **b** (column **c**), indicating absorbance characteristics that are unique for living specimens. Arrows in the difference spectra indicate species characteristic and major *in vivo* carotenoid absorption signature. *In vivo* and *in vitro* pigment signatures are primarily from carotenoids. Standard deviations are shown as bars (*N* = 3). Note different *y*-scales.

*In vivo* OD (optical density = absorbance spectra, dimensionless, OD(λ)) spectra of coral tissue indicated a pigment signature with two main absorbance peaks in all coral species examined, except for white *L. pertusa* (Figure 1a). The main peak in these samples was found from 350 to 550 nm, and OD maxima varied between 409 and 476 nm (Table 1). All species show the same OD(λ) signature at 450–550 nm, indicating the presence of astaxanthin. 

*In vitro* absorption (a(λ) g^−1^ wet weight) spectra of acetone raw pigment extract demonstrated a distinct peak in orange *L. pertusa* (Figure 1b) with a(λ_max_) at 480 nm. Absorption spectra for white *L. pertusa*, *P. arborea* and *P. resedaeformis* did not show as distinct peaks, however they showed slightly different signatures compared to the *in vivo* absorbance curves (Figure 1a). 

The *in vivo* absorption characteristics indicated differences in pigmentation between the coral species (Figure 1a). The spectral signatures from carotenoids without proteins are shown in the *in vitro* absorbance signatures (Figure 1b). The difference spectra between *in vivo* OD(λ) and *in vitro* a(λ) (Figure 1c) indicated that the signatures were species specific, illustrating spectral shifts, possibly induced by differences in pigments bound to proteins and other macro molecules. Figure 1c indicates *in vivo* absorption characteristics that are unique for the living coral specimens, indicating the presence of carotenoid-proteins. 

*In vivo* OD(λ_max_) of orange and white *L. pertusa* were found at shorter wavelengths than *in vitro* a(λ_max_) (Table 1). For *P. arborea* the *in vivo* OD(λ_max_) was found at the same wavelength as *in vitro* a(λ_max_), while for *P. resedaeformis*, *in vivo* OD(λ_max_) was found at longer wavelengths than *in vitro* a(λ_max_) (Table 1). The *in vivo* and *in vitro* coefficient of variation for mean λ_max_ was <1% for almost all species (Table 1). 

**Table 1:** Differences between *in vivo* OD and *in vitro* absorption (in acetone) maxima for all coral species. Coefficient of variation of mean value (CV%) for three replicates (*N* = 3).

		*In vivo*		*In vitro*	
Species	*N*	Mean λ_max_	CV%	Mean	CV%
Orange *Lophelia pertusa*	3	473	0.88	477	0.12
White *Lophelia pertusa*	3	409	0.61	429	0.36
*Paragorgia arborea*	3	475	1.16	475	0.76
*Primnoa resedaeformis*	3	476	0.32	463	2.27

White *L. pertusa* showed similar *in vitro* absorbance maxima as orange *L. pertusa*, although at shorter wavelengths (Figure 1b). Living specimens of orange *L. pertusa* had a broad absorption maximum from 400 to 550 nm that was not present in white *L. pertusa*. All species had an absorbance maximum at 420 nm. 

**Figure 2:**
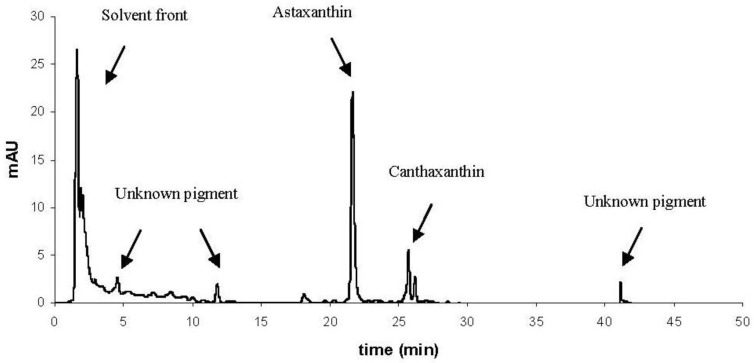
HPLC chromatogram of *Paragorgia arborea* in methanol extract, showing the main isolated pigments astaxanthin and canthaxanthin, and several unknown pigments. Solvent front contains non-separated pigments. OD (AU) detected at 440 nm.

**Figure 3:**
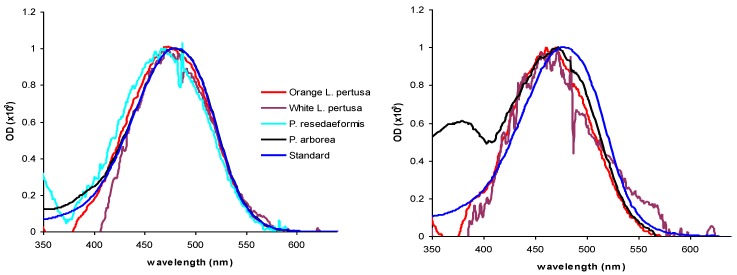
Optical density (OD) spectra from HPLC chromatograms of astaxanthin (left) and canthaxanthin (right) in all coral species studied, white and orange *Lophelia pertusa*, *Primnoa resedaeformis* and *Paragorgia arborea* in methanol extract, scaled to OD of 1. Standard of Astaxanthin and Canthaxanthin is also included for reference. Astaxanthin in *P. arborea* and orange *L. pertusa* shows the most similar absorbance as the astaxanthin reference. Note that canthaxanthin was not detected in *P. resedaeformis*.

HPLC analysis identified two major carotenoids in the corals; astaxanthin and a canthaxanthin-like carotenoid (Figure 2, Figure 3 and Table 2). Orange *L. pertusa* contained >50% astaxanthin g^−1^ wet weight compared to white *L. pertusa* (Table 3). *Paragorgia arborea* clearly contained the highest concentration of astaxanthin g^−1^ wet weight between the examined species (Table 3), and the main bulk of carotenoids were located in the epidermal layer of the coral, which is visibly the most colorful part of the coral. *Primnoa resedaeformis* contained the lowest concentration of astaxanthin, *i.e.*, <17% astaxanthin than white *L. pertusa*. In addition, HPLC chromatograms also showed non-separated pigments, detected in the solvent and for *P. arborea*: several unidentified isolated pigments at 4, 12 and 42 min (Figure 2).

**Table 2:** HPLC pigment isolation of methanol extracts of orange and white *Lophelia pertusa*, *Paragorgia arborea* and *Primnoa resedaeformis*. “X” denotes major carotenoid. Traces indicate that pigment is present in trace amount (≤1 mAU), and dash (-) indicates that pigment is not present. The main pigment, astaxanthin, appeared at 21 min, and canthaxanthin at 25 min in the HPLC chromatogram. Note that *P. arborea* contains several unknown pigments, which are not present in the other coral species.

Pigment	Retention time (min)	Orange *L. pertusa*	White *L. pertusa*	*P. arborea*	*P. resedaeformis*
Astaxanthin	21	X	X	X	X
Canthaxanthin-like	25–26	Traces	Traces	X	Traces
Unknown pigments	5, 12, 41	-	-	X	-
Pigments in solvent front	1–2	X	X	X	X

The same extracts were also subjected to LC-TOF MS analysis for determination of accurate mass of the pigments (Table 3). This analysis confirmed the main peak of orange and white *L. pertusa* and *P. arborea* from HPLC chromatograms to be astaxanthin as the measured values is within a 0.4 ppm window to the theoretical value [23]. The canthaxanthin-like carotenoid, indentified from HPLC analysis, could not be assigned accurate mass due to the small pigment concentrations in raw extracts. LC-TOF MS analysis of *P. arborea* showed high absorption in the UV region, which was not detected from *in vitro* raw extract analysis in the spectrophotometer.

**Table 3:** The main carotenoid astaxanthin isolated from deep-water corals by HPLC and assigned accurate mass from LC-TOF MS. OD maxima from HPLC OD spectra, concentration of astaxanthin from HPLC (given as ng astaxanthin ng^−1^ coral sample) and accurate molecule mass of astaxanthin from LC-TOF MS analysis, and respective ppm error.

Species	Astaxanthin			
	λ_max_ (nm) in methanol	ng astaxanthin ng^−1^ coral sample	Accurate mass (M + H)^+^	ppm error
Orange *Lophelia pertusa*	476	3.75 × 10^−7^	597.3940	0.32
White *Lophelia pertusa*	475	1.65 × 10^−7^	597.3936	−0.35
*Paragorgia arborea*	478	3.15 × 10^−6^	597.3938	0
*Primnoa resedaeformis*	473	1.37 × 10^−7^	Pigments degraded	-

Astaxanthin was the major pigment isolated from the coral samples, followed by a canthaxanthin-like carotenoid. These carotenoids are characterized with specific absorption maxima situated between 409–476 nm and 429–477 nm in the *in vivo* and *in vitro* absorbance spectra, respectively (Figure 1, Table 1). The highest concentration of astaxanthin in the corals examined was found in *P. arborea*, which is visibly the most colorful of the three coral species examined. 

From *in vitro* acetone extract analysis, indication of astaxanthin was found in orange *L. pertusa* only, as the absorption peak was distinct with a(λ_max_) at 480 nm, corresponding to astaxanthin [31]. The astaxanthin a(λ_max_) from the isolated peaks varied between coral species (Table 1), however a(λ_max_) of raw extracts were within a range of ±5 nm of the astaxanthin standard at 481 nm. The only exception to this was the a(λ_max_) of white *L. pertusa*, which deviated considerably from the astaxanthin standard. The a(λ_max_) of canthaxanthin-like carotenoid (not shown) present in raw extracts of all coral species varied between 461 nm and 475 nm, and did not fully match the absorption characteristics of the canthaxanthin standard (a(λ_max_) 476 nm). Therefore, it cannot be identified as pure canthaxanthin, but as a canthaxanthin-like carotenoid. 

HPLC confirmed the presence of significant amounts of non-extracted pigments in the solvent front which may be present as colloids in the extract (Figure 2, Table 2). These colloids or micelles may be pigments bonded to proteins, lipids and other macro molecules [32]. This is especially prominent in *P. arborea*, where the solvent front contains unidentified pigments (Figure 2). The unknown pigments were not identified from LC-TOF MS analysis, however from HPLC it was evident that two out of the three unknown pigments showed UV-A absorption (320–390 nm [33], hence this may indicate UV absorbing amino acids/proteins [23]. Orange and white *L. pertusa* showed almost identical UV absorption in the solvent front. UV absorption was also evident in *P. resedaeformis*, and UV absorption was also confirmed by LC-TOF MS analysis. It can be mentioned that other carotenoid studies of hydrocorals and sea anemones show similar results, where carotenoids have been shown to be linked to proteins [34,35]. 

Due to degradation, presumably caused by oxidation, it was not possible to confirm the presence of astaxanthin in *P. resedaeformis* raw extracts from LC-TOF MS analysis. However, it most likely contains astaxanthin, as peaks in its HPLC chromatogram appear at the same retention time as the peaks in the HPLC chromatograms of the other species studied, and the corresponding OD spectra have the same absorption characteristics. Bandaranayake (2006) [16] points out astaxanthin to be the major carotenoid in marine invertebrates, and astaxanthin in *L. pertusa*, *P. arborea* and *P. resedaeformis* has already been confirmed by Upadhyay and Liaaen-Jensen (1970) [30]. 

A high coefficient of variation of *P. arboreain vivo* and *in vitro* absorbance and *P. resedaeformisin vitro* absorbance could potentially indicate either high biological variation within the species, or insufficient extraction. Insufficient extraction may be the reason why *P. resedaeformis* contains less astaxanthin than white *L. pertusa*. A previous study by Fox and Wilkie (1970) [36] found that skeletal material in the coral *Allopora californica* did not react to common, neutral organic solvents. The pigments in this species were only released when the skeleton of the coral was exposed to acids, suggesting that astaxanthin was firmly bonded to the calcareous matrix. 

Color variation within organisms can be caused by several factors. Type of food has been known to influence coloration, as clearly shown in a study on carotenoids in salmonids [37]. *Lophelia pertusa* is likely omnivorous, feeding on a diverse range of food from zooplankton to resuspended material [38]. Copepods, which are a part of the diet of many cold water corals, contain astaxanthin, and through them, astaxanthin is transferred up the food chain [39]. However, orange and white *L. pertusa* grow side by side and have access to the same food, yet have different color and pigment concentrations. Some marine bacteria contain astaxanthin [40] and since white and orange *L. pertusa* have been shown to display different compositions of bacterial community [41,42] the color variation may be related to these differences. It might also be an inherited trait, from individual to individual, through bacteria or genetically, as the orange *L. pertusa* produce orange eggs, the white *L. pertusa* produce white eggs, although there has not been found any evidence of vertical transmission of bacteria in the eggs (Kellogg C.A. and Järnegren J. [43]). LeBoeuf *et al*. (1981) [44] suggested in a study of sea anemones that the occurrence of different colors was presumably genetically determined. It was also pointed out in a study of colors in the plumose anemone *Metridium senile* that pigmentation was not due to food, but inherited characteristics [45,46]. The association of proteins with pigments may also affect the color of the coral as indicated by this study (Figure 1). Unpublished proteomics studies by Järnegren and Collin-Hansen show a difference in protein composition between orange and white *L. pertusa*. Hence, astaxanthin is most likely not the only contributor to the orange coloration in these organisms. Given this, the variation in color may be due to bacterial or protein composition or genetic characteristics. In addition, differences in carotenoid metabolites may also be due to differences in enzymes in different color morphs of corals—another trait that may be largely due to differences in genetic composition. 

## 3. Experimental Section

### 3.1. Study Area and Sample Collection

In February 2008 and 2009, white and orange color morphs of *L. pertusa*, *P. resedaeformis* and deep red *P. arborea* were collected at Stokkbergneset 63°28′ N; 9°55′ E in the Trondheimsfjord, Norway. Corals were sampled between 100 and 200 m depth using the remotely operated underwater vehicle (ROV) “Minerva” Sperre Subfighter 7500. The ROV was operated from the research vessel Gunnerus owned by the Norwegian University of Science and Technology (NTNU). The majority of the samples were frozen immediately (−18 °C) on board the ship, while the remaining were kept alive in a flow-through aquarium (7–8 °C, *in situ* water temperature from 100 m depth) at Trondheim biological station (TBS).

### 3.2. Absorption Characteristics and Pigment Extraction

*In vivo* optical density (OD, dimensionless) was measured in a double-beam UNICAM spectrophotometer UV 500 in order to measure the absorbance characteristics in living organisms. *In vivo* tissue samples (≈200 µL) were made by crushing frozen epidermal and underlying tissue of *P. arborea* and *P. resedaeformis* in a small mortar, and adding 200 µL of filtered sea water. *Lophelia pertusa* polyps were pulled out using tweezers and placed directly on sea-water (filtered) soaked Whatman GF/C 25-mm glass fiber filters. Correspondingly, filters soaked in filtered seawater were used as blank (*N* = 3) [46,47]. To correct for scattered light, the average optical density (OD) from 750 to 800 nm was subtracted from OD(λ) 350 to 700 nm [47]. 

*In vitro* extracts were made by crushing approximately 3 g frozen coral in a mortar, in which both epidermal layer/calcareous skeleton and underlying tissue/polyps were used. To avoid pigment degradation due to higher temperatures, the mortar was placed on ice. 15 mL of acetone and methanol was added respectively and the extracts were put in glass test tubes followed by N_2_ (g) bubbling. After 24 h extraction time (−18 °C), the raw pigment extracts were sampled and filtered through a 0.2-µm filter to avoid particles and coral debris. *In vitro* absorption (m^−1^) (350 to 700 nm) of raw extracts (acetone) was measured using a 1 cm quartz cuvette in the spectrophotometer. The standard deviation between three coral samples for *in vivo* absorbance and *in vitro* absorption was calculated (*N* = 3) and from this, the coefficient of variation (CV) was calculated. *In vitro* raw pigment extracts with methanol were analyzed using High-Performance Liquid Chromatography (HPLC). 

### 3.3. Pigment Analysis

HPLC work was performed using a Hewlett-Packard 1100 series HPLC system equipped with a diode array optical density detector (350–800 nm) for pigment isolation, identification and quantification according to Rodriguez *et al*. (2006) [48]. Synthetic standards of Astaxanthin (Fluka number 32993) and Canthaxanthin (Fluka number 41659) were applied as references. 

Accurate mass determination by using an Agilent High-Performance Liquid Chromatography Time-Of-Flight Mass Spectrometer (LC-TOF MS) instrument was used as confirmatory analysis to the HPLC analyses. The extracts were run according to the protocol in Stafsnes *et al.* (2010) [49]. Acetone and methanol *in vitro* raw extracts containing minor amounts of water were used for LC-TOF MS analysis. 

## 4. Conclusions

These results indicate that astaxanthin is the major carotenoid in the deep water corals examined. Pigment composition in corals is considered as important information for chemo-taxonomy when bonded to their respective proteins (*in vivo* properties), pigment function (antibacterial), and as a basis for *in situ* optical identification for mapping and monitoring purposes. Future pigment function experiments may be of importance for bio-prospecting related to future medicine. Astaxanthin is known to be bonded to different esters and proteins giving different properties as antioxidants, immunostimulants, anti-bacterial compounds and therefore may be a major molecule group for the treatment of human illnesses, such as cancer and cardiovascular diseases [31,50,51,52,53]. Different functions of these pigment-proteins clearly need further research.
